# Low-Dose Intestinal Trichuris muris Infection Alters the Lung Immune Microenvironment and Can Suppress Allergic Airway Inflammation

**DOI:** 10.1128/IAI.01240-15

**Published:** 2016-01-25

**Authors:** Alistair L. Chenery, Frann Antignano, Kyle Burrows, Sebastian Scheer, Georgia Perona-Wright, Colby Zaph

**Affiliations:** a The Biomedical Research Centre, University of British Columbia, Vancouver, BC, Canada; b Department of Pathology and Laboratory Medicine, University of British Columbia, Vancouver, BC, Canada; c Department of Microbiology and Immunology, University of British Columbia, Vancouver, BC, Canada; d Infection and Immunity Program, Monash Biomedicine Discovery Institute, Monash University, Clayton, VIC, Australia; e Department of Biochemistry and Molecular Biology, School of Biomedical Sciences, Monash University, Clayton, VIC, Australia

## Abstract

Immunological cross talk between mucosal tissues such as the intestine and the lung is poorly defined during homeostasis and disease. Here, we show that a low-dose infection with the intestinally restricted helminth parasite Trichuris muris results in the production of Th1 cell-dependent gamma interferon (IFN-γ) and myeloid cell-derived interleukin-10 (IL-10) in the lung without causing overt airway pathology. This cross-mucosal immune response in the lung inhibits the development of papain-induced allergic airway inflammation, an innate cell-mediated type 2 airway inflammatory disease. Thus, we identify convergent and nonredundant roles of adaptive and innate immunity in mediating cross-mucosal suppression of type 2 airway inflammation during low-dose helminth-induced intestinal inflammation. These results provide further insight in identifying novel intersecting immune pathways elicited by gut-to-lung mucosal cross talk.

## INTRODUCTION

Barrier tissues such as the intestine and lungs that are exposed to the external environment are faced with the unique challenge of mounting protective immune responses against potential pathogens while maintaining homeostasis and tolerance to harmless antigens and commensal organisms. Disruption of barrier function and dysregulation of mucosal immune homeostasis are characteristic of a variety of mucosal diseases such as inflammatory bowel diseases and asthma (1–3). The mucosal immune system is interconnected, as mucosal immunization at one site can provide immunity at other mucosal sites (4, 5). This is further highlighted by the challenges of administering protective vaccines to populations that have heavy enteric pathogen burdens interfering with immunity, particularly in underdeveloped countries (6). However, little is still known about how chronic inflammation associated with infections at one barrier site influences mucosal immune responses at other sites.

Immunological cross talk can occur between distinct mucosal sites such as the lung and the intestine (7). For example, the intestinal microbiota in early life has been shown to play a role in determining susceptibility to asthma later in life (8, 9). As well, there is evidence that trafficking of immune cells can occur directly between these organs; lung dendritic cells (DCs) have the ability to prime and license lung-residing T cells to home toward intestinal tissues via specific homing receptors (10). In addition, interstitial pneumonia has been reported to occur in mouse models of chronic colitis (11) and a subset of inflammatory bowel disease patients have been reported to exhibit extraintestinal inflammation in the lungs (12). However, whether this gut-lung immune cross talk plays a physiological role in health and disease still remains unclear.

Mucosal inflammatory diseases invariably involve the action of a variety of CD4^+^ T helper (Th) cell subsets differentiating in a context-specific fashion, depending on the inflammatory milieu. Gastrointestinal inflammation often involves pathogenic Th1 or Th17 cell populations that mediate chronic inflammatory processes (13). Conversely, Th2 cells play a major role in type 2 immune responses in allergic respiratory diseases such as asthma (14, 15). Importantly, a given Th cell response can counteract the development of other Th cell responses (16), and this balance of Th cell differentiation plays a major role in determining immune homeostasis or disease susceptibility. However, this Th cell-intrinsic regulation is not as well understood in the context of mucosal immune cross talk. Further, it has recently been shown that innate cell populations, including myeloid cells and innate lymphoid cell (ILC) subsets, also play an important role during mucosal inflammation (17–19). In this study, we directly investigate the gut-lung immune axis by examining the effect of an intestinally restricted helminth infection on the lung immune microenvironment and its impact on the development of type 2 innate cell-mediated airway inflammation. We identify nonredundant, convergent adaptive, and innate immune processes by modeling this mucosal cross talk.

## MATERIALS AND METHODS

### Ethics statement.

Experiments were approved by the University of British Columbia (UBC) Animal Care Committee (ACC) (protocol number A13-0010) and were in accordance with the Canadian Guidelines for Animal Research.

### Mice and helminth infections.

C57BL/6J, GREAT [*g*amma interferon (IFN-γ) *r*eporter with *e*ndogenous poly(*A*) *t*ail] (20), and *Rag1*^−/−^ mouse strains were purchased from the Jackson Laboratory (Bar Harbor, ME). Vert-X (C57BL/6 interleukin-10 [IL-10] enhanced green fluorescent protein [eGFP]) mice were originally generated by Christopher Karp (21). All mice were bred and maintained in specific-pathogen-free animal facilities at the Biomedical Research Centre. Propagation of Trichuris muris eggs and infections were performed as previously described (22). Mice were infected with 30 hand-counted, embryonated T. muris eggs by oral gavage to induce a low-dose intestinal infection over a period of 21 days. Sacrificed mice were assessed for worm burdens by manually counting worms in the ceca using a dissecting microscope. Worm antigen was generated by pooling live worms isolated from infected immunodeficient mice into Dulbecco modified Eagle medium (DMEM) and incubating them for 4 h at 37°C. The supernatant was then dialyzed in phosphate-buffered saline (PBS) for 4 days and filter sterilized prior to quantification of total protein using a bicinchoninic acid (BCA) assay.

### Induction of acute allergic airway inflammation (AAI).

Mice were anesthetized under aerosolized isoflurane and immediately instilled with 10 μg of papain from papaya latex (Sigma, St. Louis, MO) intranasally (i.n.) in 40 μl of sterile PBS on days 18, 19, and 20 post-infection with T. muris. The day after the last instillation of papain, mice were injected intraperitoneally (i.p.) with 2,2,2-tribromoethanol (Avertin; Sigma), tracheas were cannulated, and bronchoalveolar lavages (BALs) were performed using triplicates of 1 ml of sterile 10% fetal bovine serum in PBS. BAL fluid was then red cell lysed using ammonium chloride buffer. Lung tissue was digested in 200 U/ml collagenase type IV (Sigma) for 1 h, red cell lysed, and centrifuged in a 30% Percoll solution to purify leukocytes. BAL fluid and lung cells were analyzed by flow cytometry.

### *Ex vivo* stimulation of lung cells.

Following tissue digestion and Percoll enrichment as described above, lung cells were stimulated at 37°C and 4% CO_2_ for 4 h with cell stimulation cocktail containing protein transport inhibitors (eBioscience). Cells were then processed for intracellular cytokine staining using an intracellular fixation and permeabilization buffer set (eBioscience).

### Antibodies and flow cytometry.

Cell counts were determined via hemocytometer or with latex beads for BAL fluid samples. Sample processing and intracellular cytokine staining were performed as previously described (23). Fluorescein isothiocyanate (FITC)-conjugated anti-neutrophil Ly6B (7/4) was purchased from Abcam (Cambridge, MA). Phycoerythrin (PE)-conjugated anti-Siglec F (E50-2440) and anti-CD25 (PC61) and allophycocyanin (APC)-conjugated anti-Ly6G (1A8) were purchased from BD Biosciences (San Jose, CA). FITC-conjugated anti-IL-10 (JES5-16E3); PE-conjugated anti-CD4 (RM4-4) and anti-IL-5 (TRFK4); PE-Cy7-conjugated anti-CD3e (2C11), anti-IFN-γ (XMG1.2), and anti-I-A/I-E (major histocompatibility complex class II [MHC-II]) (M5/114.15.2); peridinin chlorophyll protein (PerCP)-eFluor 710-conjugated anti-ST2 (RMST2-2); eFluor 450-conjugated anti-CD8a (53-6.7); APC-conjugated anti-CD4 (GK1.5) and anti-F4/80 (BM8); and APC-eFluor 780-conjugated anti-Ly6C (HK1.4) and anti-B220 (RA3-6B2) were purchased from eBioscience. FITC-conjugated anti-CD19 (1D3), anti-CD11b (M1/70), anti-CD11c (N418), anti-Gr1 (RB6-8C5), anti-NK1.1 (PK136), anti-Ter119 (Ter119), and anti-CD3e (2C11); Pacific Blue-conjugated anti-CD45 (I3/2) and anti-CD11b (M1/70); and Alexa Fluor 647-conjugated anti-CD11c (N418) and CD90.2 (30H12) were produced in-house by the UBC AbLab. Data were acquired on an LSR II flow cytometer (BD Biosciences) and analyzed with FlowJo software (TreeStar, San Carlos, CA).

### RNA isolation and quantitative real-time PCR (qRT-PCR).

Tissues were mechanically homogenized using a bead basher, and RNA was extracted using the TRIzol method according to the manufacturer's instructions (Ambion, Austin, TX). cDNA was generated using High-Capacity cDNA reverse transcription kits according to the manufacturer's instructions (Applied Biosystems, Foster City, CA). Quantitative PCR was performed using the SYBR FAST master mix (Kapa Biosystems, Woburn, MA) and SYBR green-optimized primer sets run on an ABI 7900 real-time PCR machine (Applied Biosystems). Threshold cycle (*C_T_*) values of all genes measured in each sample were normalized relative to beta-actin (*Actb*) gene expression. Primer sets are listed in Table 1.

**TABLE 1:** Gene expression primer sets

Primer	Sequence	
	Forward (5′–3′)	Reverse (5′–3′)
*Cd4*	CTTCGCAGTTTGATCGTTTTGAT	CCGGACTGAAGGTCACTTTGA
*Ifng*	GGATGCATTCATGAGTATTGCC	CCTTTTCCGCTTCCTGAGG
*Il5*	GATGAGGCTTCCTGTCCCTACTC	TCGCCACACTTCTCTTTTTGG
*Il10*	CTGAAGACCCTCAGGATGCG	TGGCCTTGTAGACACCTTGGTC
*Tbx21*	CAACAACCCCTTTGCCAAAG	TCCCCCAAGCAGTTGACAGT

### ELISA.

Standard sandwich enzyme-linked immunosorbent assay (ELISA) of cytokines was performed using commercially available antibodies (eBioscience). Plates were developed using 3,3′,5,5′-tetramethylbenzidine (TMB) substrate (Mandel Scientific, Guelph, ON, Canada), the reaction was stopped with 1 N HCl, and plates were read at 450 nm on a SpectraMax 384 plate reader (Molecular Devices, Sunnyvale, CA).

### Histology.

Lung lobes were fixed in buffered 4% paraformaldehyde solution and embedded into paraffin blocks. Five-micrometer-thick sections were stained with periodic acid-Schiff (PAS) stain. Airways were assessed for PAS staining as an indication of mucus hyperproduction. PAS^+^ cells in the airways in each lung section were quantified.

### Depletion of CD4^+^ T cells.

Mice were injected i.p. with 300 μg purified anti-mouse CD4 (GK1.5) in PBS or with PBS alone on days 15 and 17 postinfection, prior to airway challenge. An isotype IgG control antibody was not used since CD4 depletion creates tolerance to anti-rat Ig responses (24).

### *In vivo* neutralization of IL-10 activity.

T. muris-infected mice were injected i.p. with 500 μg purified anti-mouse IL-10 receptor (IL-10R) or control IgG1 (Bio X Cell, New Hampshire) on day 6 postinfection and then subsequently every 3 days until day 18, prior to airway challenge.

### HDM model of asthma.

For the first 3 days of airway sensitization, mice were anesthetized under aerosolized isoflurane and intranasally instilled with 100 μg of house dust mite (HDM) antigen (Greer, Lenoir, NC). On days 13 to 17 postsensitization, mice were intranasally challenged with 25 μg of HDM antigen daily before sacrifice on day 18. BAL fluid and tissues were processed as described above to assess airway disease.

### Statistics.

Data are presented as means ± standard errors of the means (SEM). Statistical significance between two groups was determined by the Student *t* test while comparisons between 3 or more groups were made by analysis of variance (ANOVA) with a Bonferroni *post hoc* test using GraphPad Prism software. Results were considered statistically significant with a *P* value of <0.05 (*), <0.01 (**), or <0.001 (***).

## RESULTS

### Low-dose T. muris infection alters the lung immune microenvironment.

**FIG 1:**
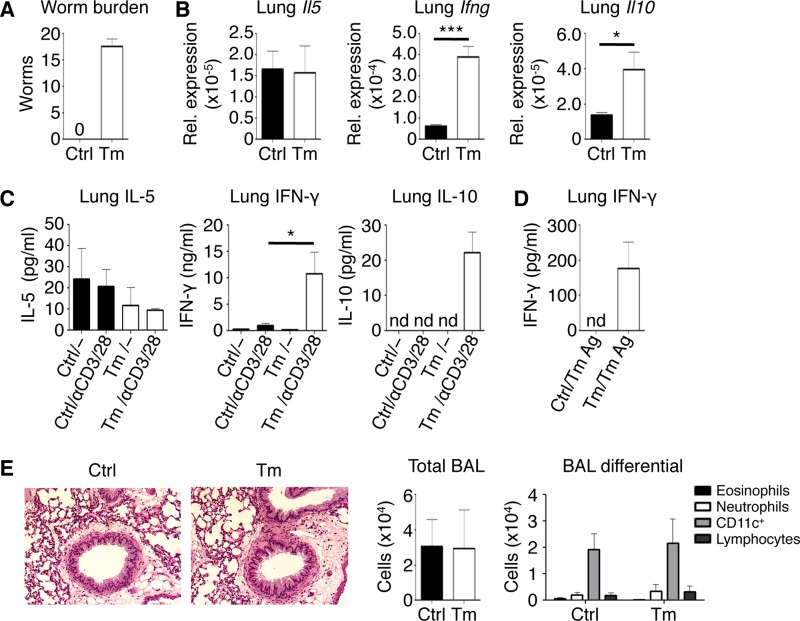
Low-dose intestinal infection with T. muris alters the lung immune microenvironment. (A) Enumerated cecal worm burdens. (B) Lung mRNA expression of *Il5*, *Ifng*, and *Il10* normalized relative to *Actb*. (C) ELISA of IL-5, IFN-γ, and IL-10 from *ex vivo* unstimulated and polyclonally stimulated lung cell cultures. (D) ELISA of IFN-γ from T. muris antigen (Ag)-stimulated lung cell cultures. (E) Lung histology visualized using ×20 magnification and total and differential bronchoalveolar lavage (BAL) fluid counts. Data are means ± SEM, pooled from 3 independent experiments (*n* = 6 mice per group) (A to E). Sections are representative of 3 independent experiments (E). Ctrl, control; Tm, T. muris infected; nd, not detected; Rel., relative.

To characterize the cross-mucosal effect of a localized intestinal immune response, we employed the Trichuris muris model of intestinal infection (25). T. muris infection is a powerful model to study the development of protective Th2 cell-dependent and nonprotective Th1 cell-mediated immune responses *in vivo*. After 21 days of a low-dose infection, C57BL/6 mice develop an adaptive anti-T. muris response, characterized by high levels of Th1 cell-derived IFN-γ, resulting in a persistent worm burden (Fig. 1A). We analyzed the distal mucosal lung tissue for changes in cytokine gene expression by qRT-PCR and found no differences in genes for Th2 cell-associated cytokines, such as *Il5*, between control and T. muris-infected mice (Fig. 1B); however, we found significantly increased expression of *Ifng* and *Il10* in the lungs of T. muris-infected mice compared to uninfected mice. These changes in cytokine gene expression in the lungs of T. muris-infected mice correlated with similar increases in IFN-γ and IL-10 protein levels when measured by ELISA of *ex vivo*-stimulated lung cell cultures (Fig. 1C). From the lungs of T. muris-infected mice, only IFN-γ, but not IL-5 or IL-10, was detectable at low levels in response to T. muris-specific antigen (Fig. 1D). Despite these changes in lung cytokine expression in T. muris-infected mice, there was no evidence of inflammation or pathology in the lungs of T. muris-infected mice compared to naive controls by histology and based on total cells and cell composition in bronchoalveolar lavage (BAL) fluid (Fig. 1E). Thus, a low-dose intestinal immune response is sufficient to alter the lung immune microenvironment by upregulating IFN-γ and IL-10 expression without causing overt lung tissue pathology.

### Lung CD4^+^ T cells increase IFN-γ production during low-dose T. muris infection.

**FIG 2:**
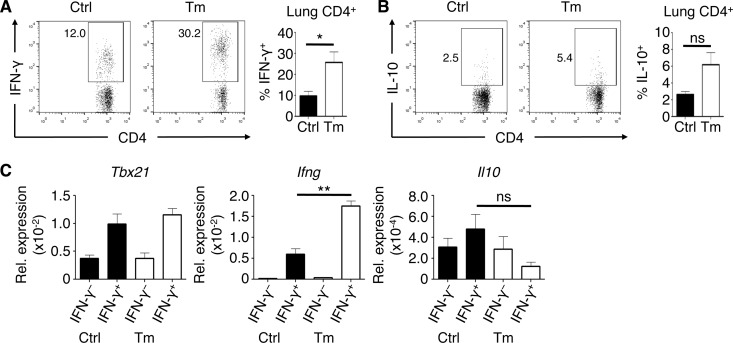
Lung CD4^+^ T cells upregulate IFN-γ expression but not IL-10 during low-dose T. muris infection. (A and B) Frequencies of CD4^+^ lymphocytes expressing IFN-γ (A) and IL-10 (B) in the lung *ex vivo* after polyclonal stimulation. (C) mRNA expression of *Tbx21*, *Ifng*, and *Il10* (relative to *Actb*) in IFN-γ^+^ and IFN-γ^−^ lung CD4^+^ T cells from GREAT mice sorted by fluorescence-activated cell sorting. Data are means ± SEM, representative of 3 independent experiments (*n* = 3 to 5 mice per experiment) (A and B) or representative of 2 independent experiments (*n* = 3 mice per experiment) (C). Ctrl, control; Tm, T. muris infected; ns, not significant; Rel., relative.

To determine the cellular source of the infection-induced IFN-γ and IL-10 in the lung, we performed *ex vivo* culture stimulations of lung cells to characterize cytokine expression by key cell populations. As several recent studies have identified populations of lung CD4^+^ T cells that produce both IFN-γ and IL-10 (26–28), we examined whether CD4^+^ T cells were the dominant source of these cytokines. Following stimulation of lung cells with anti-CD3/CD28 antibodies, we found that a significant frequency of lung CD4^+^ T cells produced IFN-γ, with the highest levels in mice infected with T. muris (Fig. 2A). In contrast, CD4^+^ T cells produced low levels of IL-10 in the lungs of either uninfected or T. muris-infected mice (Fig. 2B), and we observed a negligible population of IFN-γ/IL-10 double-positive CD4^+^ T cells (data not shown). To better assess IL-10 expression without cell stimulation, we performed fluorescence-activated cell sorting of IFN-γ^+^ and IFN-γ^−^ CD4^+^ T cells from the lungs of T. muris-infected IFN-γ reporter (GREAT) mice (20) followed by gene expression analysis *ex vivo*. We confirmed that IFN-γ-producing CD4^+^ T cells appeared to be Th1 cells that expressed *Tbx21* and *Ifng* but did not express increased levels of *Il10* (Fig. 2C). Although CD8^+^ T cells have also been shown to produce IFN-γ and IL-10 following viral infection of the lung, we failed to detect differences in CD8^+^ T cell-derived IFN-γ or IL-10 (data not shown). Together, these results show that the intestinally restricted T. muris infection results in a potent Th1 cell response in the lungs but that CD4^+^ T cells are likely not the main source of IL-10.

### Lung myeloid cells increase IL-10 expression during low-dose T. muris infection.

**FIG 3:**
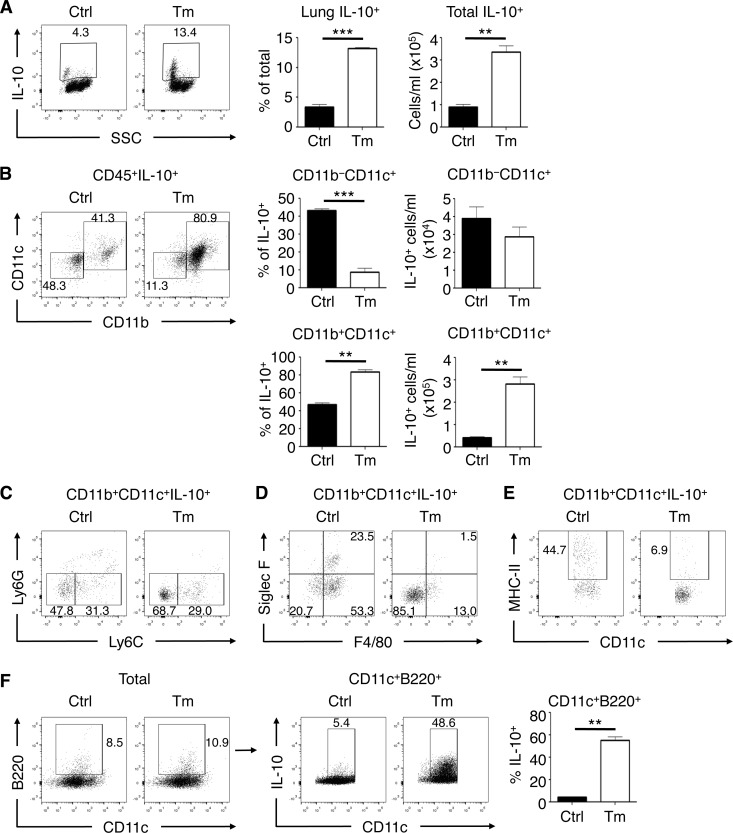
Low-dose T. muris infection induces expression of IL-10 in lung myeloid cells. (A) Total lung IL-10^+^ cell frequencies and absolute numbers in *Rag1*^−/−^ Vert-X (IL-10 reporter) mice either uninfected or infected with low-dose T. muris. SSC, side scatter. (B) Frequencies and absolute numbers of live CD45^+^-gated CD11b^−^ CD11c^+^ and CD11b^+^ CD11c^+^ cell populations comprising the total IL-10^+^ cells in the lung. (C to E) Surface marker analysis of Ly6G versus Ly6C (C), Siglec F versus F4/80 (D), and MHC-II versus CD11c (E) of CD11b^+^ CD11c^+^ IL-10^+^ gated cells. (F) Gating of total CD11c^+^ B220^+^ cells and IL-10^+^ frequencies. Data are means ± SEM, representative of 3 independent experiments (*n* = 5 to 6 mice per experiment) (A to F). Ctrl, control; Tm, T. muris infected.

Since we saw no differences in T cell-derived IL-10 levels, we hypothesized that innate cells were producing IL-10 in the lungs of T. muris-infected mice. We therefore characterized possible innate cellular sources of IL-10 induced in the lungs of T. muris-infected mice *ex vivo* using a fluorescent IL-10 reporter (Vert-X) (21) mouse strain crossed onto a *Rag1*^−/−^ background. Compared to naive control mice, T. muris-infected *Rag1*^−/−^ Vert-X mice had a significant increase in the frequency and total number of IL-10^+^ cells in the lung (Fig. 3A). There was a significant expansion of CD11b^+^ CD11c^+^ myeloid cells producing IL-10 in T. muris-infected mice compared to uninfected controls, while we saw no differences in IL-10 production between CD11b^−^ CD11c^+^ populations in terms of absolute numbers (Fig. 3B). Further phenotypic analysis showed that lung IL-10^+^ myeloid cells from T. muris-infected mice were predominantly Ly6G^−^ Ly6C^−^ (Fig. 3C) and were largely negative for surface markers associated with alveolar and interstitial macrophages such as Siglec F and F4/80 (Fig. 3D). Lung IL-10^+^ myeloid cells from T. muris-infected mice also had a reduction in MHC-II (Fig. 3E). Upon examining individual cell types for IL-10 production, we found that lung CD11c^+^ B220^+^ cells, resembling plasmacytoid DCs (pDCs), had the most significant increase in IL-10 production during T. muris infection (Fig. 3F). These data show that lung IL-10^+^ myeloid cells that are predominantly CD11b^+^ CD11c^+^, including a subset of pDC-like cells, emerge during low-dose T. muris intestinal infection, independently of adaptive immunity.

### Low-dose T. muris infection protects against innate immunity-mediated AAI.

**FIG 4:**
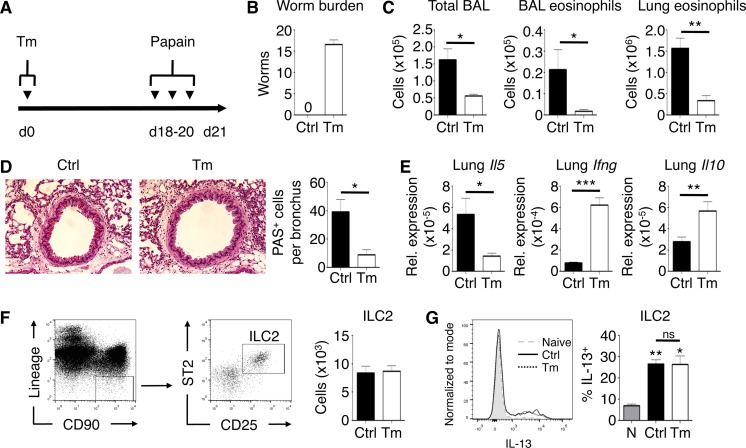
Low-dose T. muris infection suppresses papain-induced allergic airway inflammation. (A) Experimental time course of intestinal T. muris infection and airway challenge with papain. (B) Enumerated cecal worm burdens. (C) Total bronchoalveolar lavage (BAL) fluid cell, BAL fluid eosinophil, and lung tissue eosinophil counts. (D) PAS-stained sections of lungs, visualized using ×20 magnification. (E) Lung mRNA expression of *Il5*, *Ifng*, and *Il10* normalized relative to *Actb*. Rel., relative. (F) Gating of live CD45^+^ lineage^−^ CD90^+^ cells, further gated on CD25^+^ and ST2^+^ to identify ILC2 cells in uninfected or T. muris-infected mice challenged with papain with absolute numbers shown. (G) IL-13 production by ILC2 cells after *ex vivo* lung cell stimulation after papain exposure. Naïve, no papain. Data are means ± SEM, pooled from 3 independent experiments (*n* = 3 to 15 mice per group) (B to G). Sections are representative of 3 independent experiments (D). Ctrl, control; Tm, T. muris infected; ns, not significant.

We hypothesized that the immunological changes in the lung elicited by our low-dose intestinal helminth infection could affect the development of type 2 inflammatory responses, such as allergic airway inflammation (AAI). To directly address this, C57BL/6 mice were infected with a low dose of T. muris and challenged with papain intranasally (Fig. 4A). Acute airway challenge with papain causes an early activation of ILC2 cells that promotes eosinophil recruitment and mucus hyperproduction (29). Papain treatment did not affect infection dynamics, as low-dose infections persisted following papain exposure (Fig. 4B). BAL fluid analysis showed that papain induced an influx of inflammatory cells in the airways of uninfected mice (Fig. 4C). In contrast, mice infected with T. muris had a significant reduction in airway cell influx after papain challenge. While uninfected mice displayed airway eosinophilia after papain challenge, infected mice were significantly protected from eosinophil infiltration. Further, T. muris-infected mice had a reduction in lung tissue eosinophils after papain challenge. Papain-induced goblet cell hyperplasia and mucus production were also reduced in T. muris-infected mice (Fig. 4D). Expression of the Th2 cell-associated cytokine gene *Il5* in the lung was increased in control mice but was not induced in T. muris-infected mice after papain challenge, correlating with the reduction in eosinophil recruitment to the airways and lungs (Fig. 4E). Consistent with our previous results, T. muris-infected mice had significantly increased expression of lung *Ifng* and *Il10* after papain airway challenge. Despite this T. muris-mediated protection from papain-induced AAI, we found no differences in the induction of lung ILC2 cells in response to papain in terms of absolute numbers (Fig. 4F). Additionally, the capacity for lung ILC2 cells to produce IL-13 in response to papain was unaffected by T. muris infection (Fig. 4G). Thus, changes in the lung immune microenvironment during low-dose intestinal infection with T. muris are associated with protection against papain-induced AAI.

### Low-dose T. muris infection protects against acute airway inflammation in the absence of CD4^+^ T cells.

**FIG 5:**
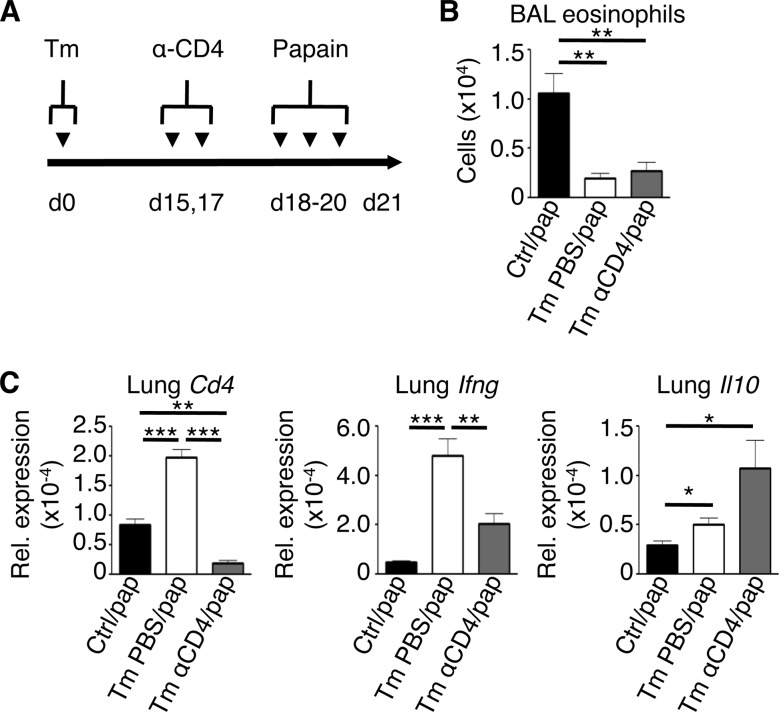
Depletion of CD4^+^ T cells does not reverse T. muris-mediated protection from papain-induced allergic airway inflammation. (A) Experimental time course of CD4 depletion during the infection/airway challenge model. (B) Bronchoalveolar lavage (BAL) fluid eosinophil counts. (C) Lung mRNA expression of *Cd4*, *Ifng*, and *Il10* (relative to *Actb*). Data are means ± SEM, representative of 2 independent experiments (*n* = 3 to 6 mice per experiment) (B and C). Ctrl, control; Tm, T. muris infected; pap, papain; Rel., relative.

Given that we saw an increase in lung CD4^+^ Th1 cells following infection with T. muris, we wanted to test whether these cells were necessary for protection against papain-induced AAI. T. muris-infected mice were treated with a depleting monoclonal antibody against CD4 prior to papain challenge (Fig. 5A). Strikingly, T. muris-infected mice depleted of CD4^+^ cells were protected from papain-induced AAI, based on a persistent reduction of airway eosinophilia compared to uninfected control mice (Fig. 5B). Gene expression analysis showed that infected, CD4-depleted mice had a significant reduction in *Ifng* expression in the lungs but still maintained increased expression of *Il10* compared to nondepleted mice (Fig. 5C). These results confirm a role for lung CD4^+^ T cells in IFN-γ production following T. muris infection and suggested that Th1 cell-expressed IFN-γ is not absolutely required for infection-induced protection against AAI. Further, these results confirm that CD4^+^ T cells are not the source of IL-10 in the lung.

### *Rag1*^−/−^ mice are partially protected from acute innate immunity-mediated airway inflammation during low-dose T. muris infection.

**FIG 6:**
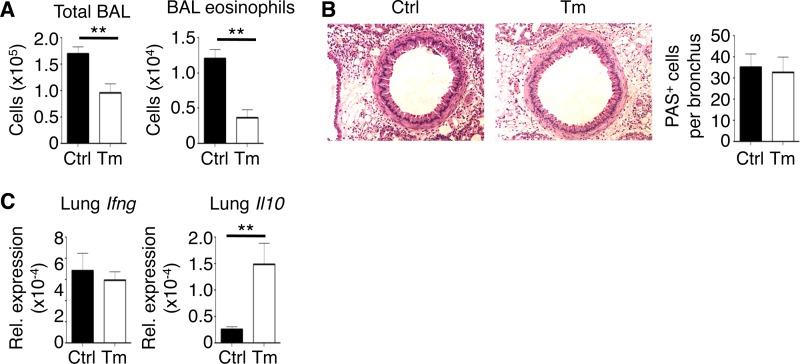
Low-dose infection with T. muris in *Rag1*^−/−^ mice partially protects from papain-induced lung inflammation. (A) Total bronchoalveolar lavage (BAL) fluid cell and BAL fluid eosinophil counts. (B) PAS-stained sections of lungs, visualized using ×20 magnification. (C) Lung mRNA expression of *Ifng* and *Il10* (relative to *Actb*). Data are means ± SEM, representative of 3 independent experiments (*n* = 4 to 5 mice per experiment) (A to C). Sections are representative of 3 independent experiments (B). Ctrl, control; Tm, T. muris infected; Rel., relative.

T. muris-infected mice that were CD4 depleted were protected from AAI despite a significant reduction in *Ifng* expression in the lungs of T. muris-infected CD4-depleted mice compared to nondepleted mice; however, lung *Ifng* levels were still elevated compared to uninfected controls, which may have represented lingering effects of IFN-γ induction in other cell types prior to CD4 depletion, as IFN-γ can exhibit positive feedback signaling (30). Therefore, we next ascertained whether the entire adaptive immune system was necessary in our experimental model. To do so, we performed our T. muris infection/papain challenge model using *Rag1*^−/−^ mice, which lack T cells and B cells but are still susceptible to innate immunity-mediated type 2 inflammation. Surprisingly, T. muris-infected *Rag1*^−/−^ mice had a significant reduction in total BAL fluid cells as well as BAL fluid eosinophils compared to uninfected *Rag1*^−/−^ mice after papain challenge (Fig. 6A). However, despite a reduction in airway eosinophilia, histopathological analysis showed that T. muris-infected *Rag1*^−/−^ mice were equally susceptible to mucus overproduction in the large airways (Fig. 6B). *Ifng* expression was not induced in the lungs of T. muris-infected *Rag1*^−/−^ mice after papain challenge, further confirming CD4^+^ T cells as the likely source (Fig. 6C). However, we observed a significant increase in lung *Il10* expression following T. muris infection, confirming an innate cell-derived source of IL-10 in the lungs during T. muris infection. These results suggest that T. muris-induced adaptive immune responses are important for regulating goblet cell hyperplasia and mucus production in the lung but are dispensable for inhibiting eosinophil infiltration into the airways after papain exposure.

### Neutralization of IL-10 activity alters disease in T. muris-infected *Rag1*^−/−^ mice during acute airway inflammation.

**FIG 7:**
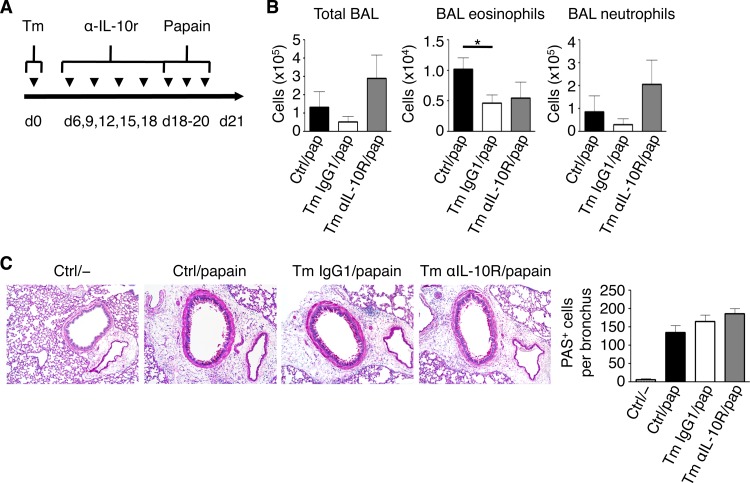
Blockade of IL-10R partially alters lung inflammation in T. muris-infected *Rag1*^−/−^ mice following papain challenge. (A) Time course of low-dose infection with T. muris and blockade of IL-10R. (B) Total bronchoalveolar lavage (BAL) fluid cell, BAL fluid eosinophil, and BAL fluid neutrophil counts. (C) PAS-stained sections of lungs, visualized using ×20 magnification. Data are means ± SEM, pooled from 2 independent experiments (*n* = 6 to 8 mice per group) (B and C). Sections are representative of 2 independent experiments (C). Ctrl, control; Tm, T. muris infected.

Since we observed a consistent upregulation of IL-10 in the lungs of T. muris-infected C57BL/6 and *Rag1*^−/−^ mice, we hypothesized that blocking IL-10 activity would restore susceptibility to eosinophilia in infected *Rag1*^−/−^ mice after papain challenge. To do so, we injected mice with anti-IL-10R antibodies every 3 days starting on day 6 postinfection, before airway challenge (Fig. 7A). Following induction of acute lung inflammation with papain, we found that compared to control and IgG1-treated T. muris-infected (T. muris IgG1) mice, anti-IL-10R-treated T. muris-infected (T. muris anti-IL-10R) mice trended toward an increase in total airway cellular infiltration as measured by total BAL fluid cell counts comprised of mainly neutrophils (Fig. 7B). However, blockade of IL-10R did not reverse suppression of eosinophilia. Consistent with our previous results, histological examination revealed no significant differences in terms of mucus hyperproduction in papain-challenged mice, with neither worm infection nor IL-10R blockade having an effect (Fig. 7C). Together, these data show that while IL-10 did not play a role in limiting eosinophil infiltration in our model, it did play a role in limiting neutrophil accumulation in the airways during acute AAI.

### Low-dose T. muris infection protects against the HDM model of murine asthma.

**FIG 8:**
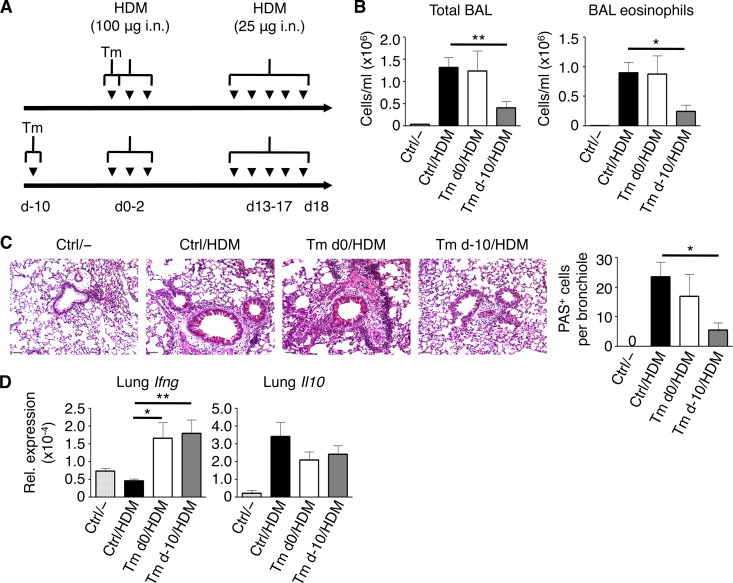
Time dependence of low-dose T. muris-mediated suppression of house dust mite (HDM) antigen-induced allergic airway inflammation. (A) Time course of low-dose infection either before (Tm d−10) or concurrent (Tm d0) with intranasal (i.n.) sensitization with HDM antigen. (B) Total bronchoalveolar lavage (BAL) fluid cell and BAL fluid eosinophil counts. (C) PAS-stained sections of lungs, visualized using ×20 magnification. (D) Lung mRNA expression of *Ifng* and *Il10* (relative to *Actb*). Data are means ± SEM, representative of 3 independent experiments (*n* = 3 to 5 mice per experiment) (B to D). Sections are representative of 3 independent experiments (C). Ctrl, control; Tm, T. muris infected; nd, not detected; Rel., relative.

We next wanted to test whether intestinal helminth infection could also abrogate the development of house dust mite (HDM)-induced AAI. HDM antigen, which displays protease activity similar to that of papain, has been used extensively as an animal model of asthma. Unlike papain, HDM-induced AAI involves priming and challenge phases of exposure to HDM antigen with a more prominent role for adaptive immune responses. Thus, we infected C57BL/6 mice with T. muris either 10 days before the sensitization phase (T. muris d−10) or on the first day of sensitization (T. muris d0) (Fig. 8A). We found that after challenge with HDM antigen, control and T. muris d0 mice had an increase in total BAL fluid cells and eosinophils while T. muris d−10 mice had significantly fewer total cells and eosinophils in the BAL fluid (Fig. 8B). Histologically, control and T. muris d0 mice after HDM antigen challenge had an increase in cellular infiltration around airways as well as evidence of mucus hyperproduction in bronchioles; in contrast, the lungs of T. muris d−10 mice largely resembled naive lungs with a reduction in both airway cellular infiltration and mucus production (Fig. 8C). Gene expression analysis of the lungs showed a significant increase in *Ifng* expression in both groups of T. muris-infected mice following HDM antigen challenge (Fig. 8D); there were no significant differences found between control mice and infected mice in terms of *Il10* gene expression. Based on these results, we conclude that T. muris-mediated protection from HDM antigen-induced airway disease likely involves an effect during the early sensitization phase of the model.

## DISCUSSION

Low-dose infections with T. muris cause a localized intestinal Th1 cell response, resulting in parasite persistence in most strains of wild-type mice (25). Since low-dose T. muris infections have been shown to induce increased intestinal levels of IFN-γ and IL-10 (31, 32), we assessed whether levels of these cytokines were also affected distally in the lungs. We found that T. muris infection was sufficient to significantly increase IFN-γ and IL-10 expression in the lung, in the absence of any signs of airway pathology. Our follow-up analyses determined that T. muris infection-induced lung IFN-γ was predominately produced by CD4^+^ Th1 cells while IL-10 was mainly myeloid cell derived. Furthermore, these infection-induced changes in the lung immune microenvironment correlated with complete resistance to innate immunity-mediated type 2 responses to the protease allergen papain.

Our results suggest that both adaptive and innate responses were required for mediating infection-induced distal suppression of AAI. IFN-γ from Th1 cells may inhibit the development of Th2 cell responses during AAI, but its role has been controversial and context dependent (33–35). It has been reported previously that IFN-γ can negatively regulate goblet cell function during AAI (36, 37), a role that is consistent with our results showing a consistent infection-induced upregulation of lung IFN-γ correlating with reductions in goblet cell-derived mucus production during AAI. In parallel, IL-10 has pleiotropic effects on all immune cells and, during AAI, can be considered to be protective (38, 39); for example, IL-10 can regulate Th2 cell cytokine responses and have a negative effect on eosinophil function and survival (40–42). To tease out the individual roles of these cytokines, we showed that T. muris-infected *Rag1*^−/−^ mice, which had elevated lung IL-10 in the absence of lung IFN-γ responses, were not fully protected from AAI. In addition, we blocked IL-12, a cytokine that potently drives IFN-γ expression, and found that this was not sufficient to reverse protection from AAI during T. muris infection (see Fig. S1 in the supplemental material). Thus, infection-induced IFN-γ and IL-10 have nonredundant roles in the inhibition of AAI.

Previously, Mohrs et al. showed that infection with a different intestinal helminth, Heligmosomoides polygyrus bakeri, which induces a mixed Th2/regulatory T (Treg) cell response, results in systemic dissemination of Th2 cells to extraintestinal sites, including the airways (43); together with our results showing accumulation of Th1 cells in the lung following T. muris infection, this suggests that specific Th cell-polarized responses can propagate from the intestine to the lung. In parallel with this Th1 response, the innate IL-10^+^ cells may represent a heterogeneous population of immature myeloid cells that expand during low-dose T. muris infection. Further, our results demonstrate that a prominent subset of these myeloid cells were B220^+^, resembling plasmacytoid DCs (pDCs); pDCs have been associated with the priming of IL-10-producing Treg cells (44) but have not been described before to intrinsically produce IL-10. In the context of cross-mucosal trafficking, lung DCs can upregulate gut-homing receptors on T cells to induce migration from the lung to the gut (10). Given that we saw T. muris antigen-responding Th1 cells in the lung, T cell migration between the gut and the lung may actually be bidirectional. However, it remains to be determined whether these adaptive and innate cells are intestinally derived cells that traffic to the lung or are instead locally induced *de novo* in the lung mucosa following systemic dissemination of helminth antigens. Additionally, future studies should determine whether T. muris infection affects hematopoiesis in the bone marrow, since IFN-γ is known to profoundly impact hematopoietic progenitor cells and eosinophil differentiation (45).

It has previously been shown that intestinal *H. polygyrus* infection can also suppress murine models of asthma, primarily through the induction of Treg cells via a helminth-secreted transforming growth factor beta (TGF-β) analog (46). Further, soluble excretory/secretory products from *H. polygyrus*, when coadministered with allergens intranasally, are sufficient in suppressing type 2 responses (47). In contrast, we found that our model of intestinal T. muris infection modulating lung immune responses does not depend on Treg cell responses when considering our CD4 depletion results and after quantifying lung Treg cells following papain exposure (see Fig. S2 in the supplemental material). *H. polygyrus*-mediated suppression of AAI is dependent on IL-10 (48); although we found a consistent upregulation of IL-10 in the lung during T. muris infection, innate IL-10 in infected *Rag1*^−/−^ mice was not sufficient to fully protect against AAI. Instead, our results suggest nonredundant roles for both Th1 cells and innate myeloid cells in T. muris infection-mediated protection from type 2 airway inflammation. Thus, we hypothesize that the Th1 cell-associated response to T. muris gets misdirected to the lungs and, in the absence of appropriate antigen, becomes self-limiting by inducing immunoregulatory responses involving IL-10, resulting in bystander immunosuppression in the local airway mucosa. In parallel to these responses, it has been shown that chronic T. muris infection causes alterations in the intestinal microbiota composition and metabolome (49) which may potentially play a role in mediating gut-to-lung immune cross talk, since this has been described to occur during *H. polygyrus* infection (50).

Finally, we assessed the impact of our Th1 cell-driven infection model on an AAI model more relevant to human asthma, with a significant adaptive immune component. Complete protection from HDM antigen-induced AAI occurred only in mice infected with T. muris prior to sensitization, suggesting that the timing of the infection is critical in establishing protection against asthma. Consistent with our data on early type 2 responses to papain, this likely involves a critical effect of the infection on the early phases of type 2 inflammation. This may partially explain why treatment of allergic rhinitis patients with Trichuris suis ova has been found to be ineffective during the peak of allergic symptoms (51). Broadly, this suggests that Th1 cell-driven intestinal inflammation and associated immunoregulatory responses are effective only as a prophylactic means to suppress type 2 responses, as opposed to being therapeutic.

In summary, we have shown that an intestinally restricted, Th1 cell-driven infection can potently suppress the development of innate immunity-mediated type 2 responses in the lung, in a temporally dependent manner. The cellular mechanisms involved display a complex, multifaceted means of host immunomodulation that involves both innate and adaptive immunity and spans distinct mucosal tissues. These converging immune pathways may highlight novel avenues of better understanding cross-mucosal immune regulation.

## Supplementary Material

Supplemental material
