# Supplementary material for: Low-Dose Intestinal Trichuris muris Infection Alters the Lung Immune Microenvironment and Can Suppress Allergic Airway Inflammation
Source: Infect Immun. 2016 Jan 25;84(2):491–501. doi: 10.1128/IAI.01240-15 (PMC4730564; doi:10.1128/IAI.01240-15)
Supplement: Supplemental material [file supp_84_2_491__index.html]

Supplemental material 

# Low dose intestinal *Trichuris muris* infection alters the lung immune microenvironment and can suppress allergic airway inflammation

## Supplemental material

- Supplemental file 1 -

  Fig. S1. Neutralization of IL-12 does not reverse *T. muris* infection-mediated protection from papain-induced allergic airway inflammation. Fig. S2. *T. muris* infection does not affect lung Treg cells.

  PDF, 143K
