# Supplementary material for: Low-Dose Intestinal Trichuris muris Infection Alters the Lung Immune Microenvironment and Can Suppress Allergic Airway Inflammation
Source: Infect Immun. 2016 Jan 25;84(2):491–501. doi: 10.1128/IAI.01240-15 (PMC4730564; doi:10.1128/IAI.01240-15)
Supplement: Supplemental material [file IAI.01240-15_zii999091574so1.pdf]

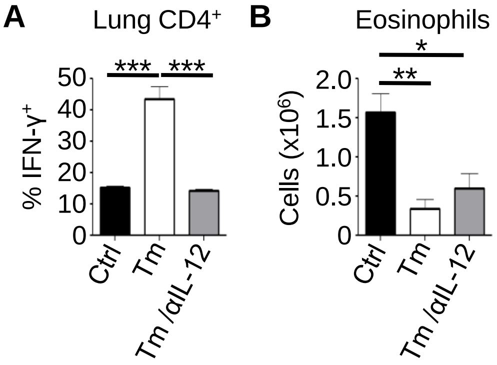

FIG. S1. Neutralization of IL-12 does not reverse *T. muris* infection-mediated protection from papain-induced allergic airway inflammation. (A) *Ex vivo* intracellular staining of IFN-γ from lung $CD4^+$ cells following papain exposure. (B) Quantification of total lung eosinophils following papain exposure. Data are means ± SEM, representative of 2 independent experiments, n= 3 mice per experiment (A-B). (Ctrl: papain/IgG1 treated; Tm: *T. muris*-infected papain/IgG1 treated; Tm/αIL-12: *T. muris*-infected/anti-IL-12 treated/papain treated).

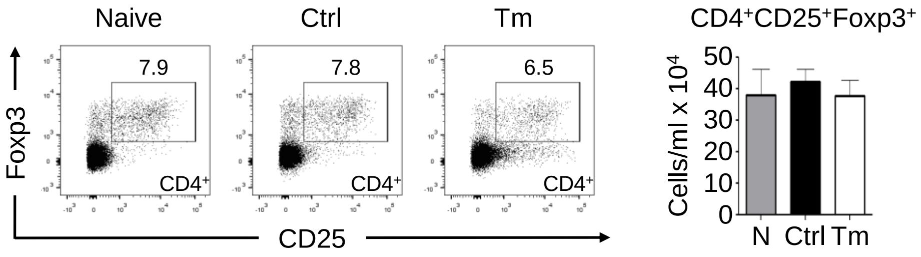

FIG. S2. *T. muris* infection does not affect lung Treg cells. $CD4^+$-gated frequencies and absolute numbers of lung $Foxp3^+CD25^+$ Treg cells after Tm infection and papain exposure. Data are means ± SEM, representative of 2 independent experiments, n= 3 mice per experiment. (N: naive, no papain; Ctrl: papain treated; Tm: *T. muris*-infected papain treated).
